# *MCM9* is associated with germline predisposition to early-onset cancer—clinical evidence

**DOI:** 10.1038/s41525-021-00242-4

**Published:** 2021-09-23

**Authors:** Yael Goldberg, Ola Aleme, Lilach Peled-Perets, Sergi Castellvi-Bel, Maartje Nielsen, Stavit A. Shalev

**Affiliations:** 1 Raphael Recanati Genetic Institute, Rabin Medical Center—Beilinson Hospital, Petah Tikva, Israel; 2 Sackler Faculty of Medicine, Tel Aviv University, Tel Aviv, Israel; 3 Genetic Institute, HaEmek Medical Center, Afula, Israel; 4 Gastroenterology Department, Institut d’Investigacions Biomèdiques August Pi i Sunyer (IDIBAPS), Centro de Investigación Biomédica en Red de Enfermedades Hepáticas y Digestivas (CIBERehd), Hospital Clínic, Barcelona, Spain; 5 Department of Clinical Genetics, Leiden University Medical Center, Leiden, The Netherlands; 6 Rappaport Faculty of Medicine, Technion—Israel Institute of Technology, Haifa, Israel

**Keywords:** Cancer genetics, Molecular medicine

## Abstract

Mutated *MCM9* has been associated with primary ovarian insufficiency. Although *MCM9* plays a role in genome maintenance and has been reported as a candidate gene in a few patients with inherited colorectal cancer (CRC), it has not been clearly established as a cancer predisposition gene. We re-evaluated family members with *MCM9-*associated fertility problems. The heterozygote parents had a few colonic polys. Three siblings had early-onset cancer: one had metastatic cervical cancer and two had early-onset CRC. Moreover, a review of the literature on *MCM9* carriers revealed that of nine bi-allelic carriers reported, eight had early-onset cancer. We provide clinical evidence for *MCM9* as a cancer germline predisposition gene associated with early-onset cancer and polyposis, mainly in a recessive inheritance pattern. These observations, coupled with the phenotype in knockout mice, suggest that diagnostic testing for polyposis, CRC, and infertility should include *MCM9* analysis. Early screening protocols may be beneficial for carriers.

## Introduction

Identification of high-risk polyposis and colorectal cancer (CRC) predisposition genes is imperative in order to prevent CRC in carriers and their relatives. The estimated heritability of CRC ranges from 16 to 35%^1^. Genetic predisposition to CRC has been classically associated with germline mutations or, in nonpolyposis cases, epimutations in the DNA mismatch repair (MMR) genes. Genes associated with dominant polyposis syndromes include *APC* (FAP MIM #175100), *POLE* and *POLD1* (PPAP MIM #615083 and 612591), *GREM1* (HMPS1 MIM #601228), *SMAD4* (JPS MIM #175050), *BMPR1A* (JPS MIM #174900), *STK11* (PJS MIM #175200), and *PTEN* (MIM #158350). Recessive inheritance of polyposis has been associated with mutations in *MUTYH* (MAP MIM #608456) and *NTHL1* (NAP MIM #616415) and the MMR genes (CMMRD MIM #276300). Recently, *APC* mosaicism was added as a relatively common cause of polyposis in isolated cases^2^. However, only 4–8% of all patients with CRC are presently found to carry germline pathogenic variants in one of the known high-penetrance genes^3^, and overall, a considerable proportion of familial aggregation of CRC remains unexplained^3^. Notably, most cancer predisposition conditions described today are non-syndromic.

The minichromosome maintenance 9 homologous recombination repair factor gene *MCM9* (MIM #610098) functions together with *MCM8* in a helicase hexameric complex involved in genome maintenance, meiotic recombination, and repair of double-strand breaks via homologous recombination^4^. The *MCM8/9* complex is required for proper localization of the MRN (Mre11-Rad50-Nbs1) complex to DNA double-strand breaks and for DNA resection to enable homologous recombination^5,6^. Cells lacking *MCM8/9* are viable but highly sensitive to inter-strand cross-linking-inducing agents, and they exhibit more chromosome aberrations in the presence of mitomycin C than wild-type cells^7^. *MCM9* is apparently also required for mammalian MMR^8^ as it co-immunoprecipitates with *MCM8*, *MSH2*, and *MLH1*, and its deficiency has been linked to microsatellite instability (MSI) and MMR alterations^8^.

*MCM9* knockout mice are viable but undergo embryonic germ cell depletion with production of a reduced quantity of spermatozoa due to defective stem-cell renewal. *MCM9* impairment induces meiotic recombination defects and oocyte degeneration^4^. Female mice are sterile, with ovaries that are devoid of oocytes. *MCM9* knockout mice were found to be at high risk of hepatocellular carcinomas and ovarian tumors^4,9^.

We and others have described several families in which bi-allelic mutations in *MCM8* or *MCM9* were associated with primary ovarian insufficiency (POI)^7,10–15^. Early-onset polyposis and CRC were reported in only one of them^10^ (Table 1). Very recently, Golubicki et al.^16^ reported two patients with CRC who had bi-allelic *MCM9* mutations*;* one also had POI (Table 1). Nevertheless, *MCM9* has not been defined as a cancer predisposition gene^17,18^ and is not tested in targeted panels.

**Table 1:** *MCM9* carriers with reported polyposis and/or cancer.

Study	Subject ID in publication	Variant	Zygosity	Sex	Polyps, no, (age at diagnosis)	Cancer (age at diagnosis)	MMR status	Fertility (age at diagnosis)
Current	III-3	c.1483G > T, (p.E495*)	Mono-allelic	F	Yes, 3 (68)	No		Normal
	III-2	c.1483G > T, (p.E495*)	Mono-allelic	M	Yes, 3 (66–68)	No		Normal
	IV-1	c.1483G > T, (p.E495*)	Bi-allelic	F	Yes (NA)	CRC (31)	NT	POI (15)
	IV-2	c.1483G > T, (p.E495*)	Bi-allelic	F	NT	Clear cell carcinoma of cervix (37)	NT	POI (15)
	IV-3	c.1483G > T, (p.E495*)	Mono-allelic	M	Yes (NA)	CRC (35)	MSS	OTA
Goldberg et al.^10^	II-1	c.672_673delGGinsC, (p.Glu225Lysfs*4)	Mono-allelic	F	Yes, >10 (53–65)	No		Normal
	II-2	c.672_673delGGinsC, (p.Glu225Lysfs*4)	Mono-allelic	M	Yes, 1 (83)	CRC (83)	MSS	Normal
	III-3	c.672_673delGGinsC, (p.Glu225Lysfs*4)	Bi-allelic	F	Yes, >40 (34)	CRC (34)	MSS	POI (<20)
	III-4	c.672_673delGGinsC, (p.Glu225Lysfs*4)	Bi-allelic	F	Yes >20 (37)	CRC X 2 (37)	NT	POI (<20)
	III-1	c.672_673delGGinsC, (p.Glu225Lysfs*4)	Mono-allelic	F	No	No		Normal
	III-2	c.672_673delGGinsC, (p.Glu225Lysfs*4)	Mono-allelic	F	Yes, 2 (39)	No		Normal
Golubicki et al.^16^	011-69294-1	c.1642C > T, p.(Arg548Trp) c.152A > T, p.(Asn51Ile)	Bi-allelic	F	Yes (>40)	CRC (45)	MSI	POI (<40)
	011-69294-2	c.1642C > T, p.(Arg548Trp) c.152A>T, p.(Asn51Ile)	Bi-allelic	M	Yes (>40)	CRC (55)	MSS	NA
	MSS13-1961	c.3425A > G, p.(Lys1142Arg); c.1640T > C, p.(Leu547Pro)	Bi-allelic	F	No	CRC (42)	MSS	
Alvarez-Mora et al.^13^	POI_02	c.1473dupT (p. T492Yfs*4)	Bi-allelic	F	NT	germ cell tumor (14)	NT	POI (20)
	POI_03	c.1473dupT (p. T492Yfs*4)	Bi-allelic	F	NT			POI (14)

All ages reported in years.

*CRC* colorectal cancer, *MSI* microsatellite instability, *MSS* microsatellite stability, *NA* not available, *NT* not tested, *OTA* oligoteratoasthenozoospermia, *POI* primary ovarian insufficiency.

Prompted by these findings, we re-evaluated a family with an *MCM9* pathogenic variant, previously evaluated at our medical center for POI, and revealed that three siblings had early-onset cancer.

## Results and discussion

**Fig. 1:**
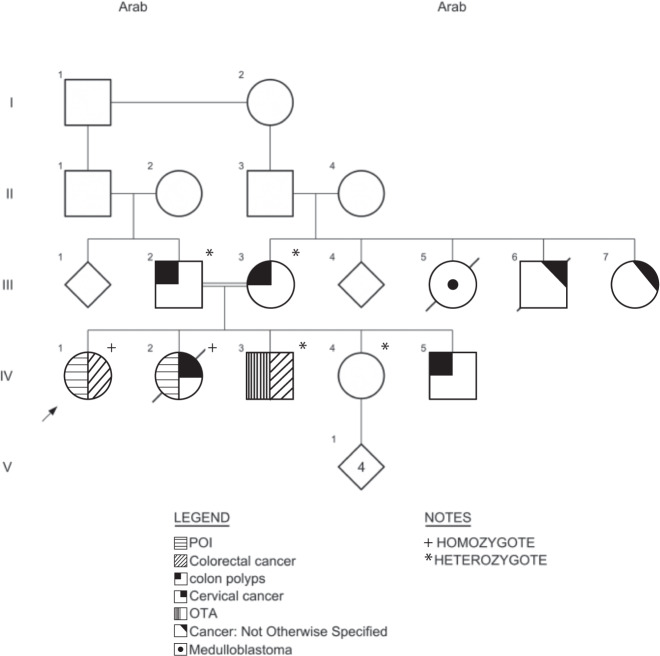
*MCM9* mutated pedigree. Clinical manifestations, polyps, and cancers are indicated. Genotypes are indicated as: + for homozygotes; * for heterozygotes. Tumor spectrum included cancer—not otherwise specified, cervical cancer, colon polyps, colorectal cancer, medulloblastoma, oligo-terato-asthenozoospermia (OTA), and primary ovarian insufficiency (POI).

The clinical and pathological data of the family described in the present study are shown in Table 1 and Fig. 1.

The parents (III-2, III-3) are first paternal cousins of Middle Eastern Arabic origin. They have five children, three daughters (IV-1, IV-2, IV-4) and two sons (IV-3, IV-5). Two daughters were diagnosed with POI around age 15 years. Karyotype was normal (46, XX), and there was no premutation in the *FMR1* gene. Both were infertile. One of the two sons was diagnosed with severe oligoteratoasthenozoospermia (OTA) at age 35 years. Whole-exome sequencing detected the c.1483G *>* T [p.E495*] variant in the *MCM9* gene^14^.

The c.1483G *>* T [p.E495*] variant introduces a premature stop codon in coding exon 8 and is expected to lead to the loss of a functional protein. The variant is not found in gnomAD exomes and genomes. The pathogenic computational verdict is based on five pathogenic predictions. Segregation analysis indicated autosomal recessive inheritance in the family.

Re-evaluation of the family for the present study revealed that three of the siblings had cancer and the parents had a few colonic polyps. Current clinical and pathological data are shown in Fig. 1 and Table 1.

Patient IV-1, homozygous for the *MCM9* variant, is currently 40 years old. She was diagnosed at age 15 years with primary amenorrhea secondary to hypogonadism, and was treated with hormonal replacement. At age 31 years, colonoscopy performed to evaluate rectal bleeding revealed a villous adenoma with high-grade dysplasia and foci of well-differentiated adenocarcinoma.

Patient IV-2, homozygous for the *MCM*9 variant, was, like her sister, diagnosed with primary amenorrhea around age 15 years. At age 37 years, she was found to have metastatic clear cell carcinoma of the cervix. PET-CT scan showed massive involvement. She died 2 months after diagnosis.

Patient IV-3, heterozygous brother, was diagnosed with OTA. Karyotype, Y micro-deletions, and *CFTR* gene testing were normal. At age 35 years, he presented with rectal bleeding and weight loss and was ultimately diagnosed with moderately differentiated colon adenocarcinoma with liver involvement. Findings on immunohistochemistry for the four MMR proteins were normal. Surveillance revealed recurrent polyps.

Patient IV-4, heterozygous for the *MCM9* variant, is healthy at age 42 years. She has four healthy children and did not report any fertility problems. She has not undergone colonoscopy.

Patient IV-5 has never gone for genetic counseling but has recurrent polyps.

Both parents (III-2 and III-3) are cancer free at age 69 and 74 years. They did not report fertility problems. Colonoscopy performed in the mother at age 68 years for evaluation of rectal bleeding revealed three polyps. The father had one tubular adenoma at age 66 years and one tubulo-villous adenoma with low-grade dysplasia and one hyperplastic polyp at age 68 years. The family history was remarkable for early cancer in three family members, one with suspected medulloblastoma and one reported to have died of an “abdominal tumor” (Fig. 1).

### Updated review of published cases

Our review of previous publications on *MCM9* carriers from families with cancer (Table 1) yielded 11 additional patients. Of the nine bi-allelic carriers, eight reported fertility problems and eight had early-onset cancer, including six with CRC (ages 31–55 years; mean 41). Three of the four tumors analyzed for MSI were stable. Multiple polyps were documented in four bi-allelic carriers, and gynecologic cancer was reported in two bi-allelic women (ages 14 and 37 years). Among the seven mono-allelic carriers, two (ages 35 and 83 years) had CRC and six (ages 39–83 years) had a few colonic polyps.

This report complements our previous description^14^ and, together with the work of Golubicki et al.^16^, supports the recognition of *MCM9* as a cancer predisposition gene associated with early-onset cancer, mainly CRC and gynecologic tumors, in addition to infertility. The mode of inheritance is autosomal recessive, with some evidence of a milder mono-allelic, phenotype.

The co-occurrence of infertility and early-onset cancer, both conditions associated with mutated *MCM9*, in the heterozygote brother (Patient IV-3) is intriguing because it may indicate substantial malfunction of *MCM9* also in the mono-allelic state. A heterozygous variant in *MCM9* leading to Y511* was reported in ClinVar in a patient with POI (ClinVar 156586), and in three MCM heterozygotes with POI^15^. Moreover, *MCM8-9* heterozygous variants were identified in 16/131 Dutch cases of polyposis and familial cancer^16^, and CRC and a few polyps at a later age were reported in heterozygote parents^10^. Thus, mono-allelic carriers may be at increased risk, with low penetrance or late onset. The effect of *MCM9* mutations might be dosage-dependent whereas bi-allelic variations lead to more severe defects.

However, given the relative high frequency of both infertility and cancer in the general population, incidental irrelevant *MCM9* heterozygous findings could be argued.

Of the four tumors analyzed for MSI, three were stable. MCM9 is required for mammalian MMR^18^. *MCM8/9* deficiency impairs HR-mediated DNA repair, and deficient cells are particularly sensitive to DNA cross-linking agents and inhibition of poly-ADP ribose polymerase^19,20^. This might vindicate both MSI signatures.

To conclude, we provide clinical supporting evidence for *MCM9* as a cancer germline predisposition gene associated with early-onset CRC and polyposis, mainly in a recessive inheritance pattern. The new data presented here, by itself not sufficient to conclude that MCM9 is causal for either polyposis or CRC, however, these observations, coupled with the phenotype in knockout mice^10,16^, suggest that early screening protocols may be beneficial for carriers. Diagnostic testing for polyposis, CRC, and infertility should include analysis of *MCM9*. Additional studies in other cohorts including patients affected with familial cancer, colonic polyposis, and/or fertility problems could help to further define the role of *MCM9* in predisposing to these pathologies.

## Methods

### Background

In 2016, a report from the HaEmek Medical Center in Israel, by Fauchereau et al.^14^ described the genetic findings in a consanguineous family with POI. Whole-exome sequencing of two patients and a non-affected sister yielded a homozygous causal variant in the *MCM9* gene, c.1483G > T [p.E495*], apparently leading to loss of a functional protein.

### Patient re-evaluation

For the present study, to investigate the possible association among mutant *MCM9*, infertility, and a predisposition to cancer, we retrieved the clinical and pathological data from the files of the patients evaluated by Fauchereau et al.^14^ at our medical center 5 years previously. We searched for additional morbidities and assessed the clinical, oncologic, and pathologic findings. All patients described in this manuscript provided written informed consent. The study was approved by the HaEmek Medical Center Research Ethics Committee.

### Literature review

Public resources such as Pubmed and ClinVar were searched for publications on *MCM9* carriers using the keyword “MCM9”. Data on cancer and colonic polyps among *MCM9* carriers and/or family members were retrieved and analyzed.

### Reporting summary

Further information on research design is available in the Nature Research Reporting Summary linked to this article.

## Supplementary information

Reporting Summary

## Data Availability

The authors declare that data supporting the findings of this study are available within the paper. Further information may be found in the online version of the original article describing the genetic analysis of the patients (family MO4)^14^ https://onlinelibrary.wiley.com/action/downloadSupplement?doi=10.1111%2Fcge.12736&file=cge12736-sup-0003-AppendixS1.pdf. All other data are available on reasonable request.
